# Circadian rhythms in septic shock patients

**DOI:** 10.1186/s13613-021-00833-5

**Published:** 2021-04-26

**Authors:** Gunnar Lachmann, Bharath Ananthasubramaniam, Viktor A. Wünsch, Lara-Marie Scherfig, Clarissa von Haefen, Cornelia Knaak, Andreas Edel, Lukas Ehlen, Barbara Koller, Anton Goldmann, Hanspeter Herzel, Achim Kramer, Claudia Spies

**Affiliations:** 1 Department of Anesthesiology and Operative Intensive Care Medicine (CCM, CVK), Charité–Universitätsmedizin Berlin, Augustenburger Platz 1, 13353 Berlin, Germany; 2 Berlin Institute of Health (BIH), Anna-Louisa-Karsch-Str. 2, 10178 Berlin, Germany; 3 Institute for Theoretical Biology, Charité–Universitätsmedizin Berlin, Berlin, Germany; 4 Laboratory of Chronobiology, Charité–Universitätsmedizin Berlin, Berlin, Germany; 5 Department for Neurology and Neurological Intensive Care Medicine, Asklepios Fachklinikum Brandenburg, Brandenburg an der Havel, Deutschland; 6 Humboldt Universität zu Berlin, Unter den Linden 6, 10117 Berlin, Germany

**Keywords:** Clock genes, Septic shock, Sepsis, Circadian rhythm, Pathophysiology

## Abstract

**Background:**

Despite the intensive efforts to improve the diagnosis and therapy of sepsis over the last decade, the mortality of septic shock remains high and causes substantial socioeconomical burden of disease. The function of immune cells is time-of-day-dependent and is regulated by several circadian clock genes. This study aims to investigate whether the rhythmicity of clock gene expression is altered in patients with septic shock.

**Methods:**

This prospective pilot study was performed at the university hospital Charité–Universitätsmedizin Berlin, Department of Anesthesiology and Operative Intensive Care Medicine (CCM, CVK). We included 20 patients with septic shock between May 2014 and January 2018, from whom blood was drawn every 4 h over a 24-h period to isolate CD14-positive monocytes and to measure the expression of 17 clock and clock-associated genes. Of these patients, 3 whose samples expressed fewer than 8 clock genes were excluded from the final analysis. A rhythmicity score S_P_ was calculated, which comprises values between -1 (arrhythmic) and 1 (rhythmic), and expression data were compared to data of a healthy study population additionally.

**Results:**

77% of the measured clock genes showed inconclusive rhythms, i.e., neither rhythmic nor arrhythmic. The clock genes *NR1D1*, *NR1D2* and *CRY2* were the most rhythmic, while *CLOCK* and *ARNTL* were the least rhythmic. Overall, the rhythmicity scores for septic shock patients were significantly (*p* < 0.0001) lower (0.23 ± 0.26) compared to the control group (12 healthy young men, 0.70 ± 0.18). In addition, the expression of clock genes *CRY1*, *NR1D1*, *NR1D2*, *DBP*, and *PER2* was suppressed in septic shock patients and *CRY2* was significantly upregulated compared to controls.

**Conclusion:**

Molecular rhythms in immune cells of septic shock patients were substantially altered and decreased compared to healthy young men. The decrease in rhythmicity was clock gene-dependent. The loss of rhythmicity and down-regulation of clock gene expression might be caused by sepsis and might further deteriorate immune responses and organ injury, but further studies are necessary to understand underlying pathophysiological mechanisms.

*Trail registration* Clinical trial registered with www.ClinicalTrials.gov (NCT02044575) on 24 January 2014.

**Supplementary Information:**

The online version contains supplementary material available at 10.1186/s13613-021-00833-5.

## Background

Sepsis is estimated to affect more than 19 million people worldwide every year and might cause up to 5 million deaths [1]. In adult intensive care units (ICUs), sepsis and septic shock are the leading causes of death [2]. Even though multiple studies substantially improved the understanding the complex pathophysiology of sepsis, several treatments have failed in clinical trials [2]. Nevertheless, with rising rates of overall incidence and fatal mortality, there is an urgent need towards targeted sepsis therapy addressing individual disease trajectories [3].

Conventionally, sepsis is thought to be the result of an exaggerated, hyperinflammatory response. However, current data indicate more complex mechanisms, such as a suppressed inflammatory response, metabolic changes and dysregulated coagulation [4]. A dysregulated immune response leads to the formation of systemic inflammatory response syndrome (SIRS) and compensatory anti-inflammatory response syndrome (CARS) at the same time [5]. The further course of sepsis is presented as either a recovery to immune homeostasis, or the persistence of CARS with severe immunosuppression [5–7]. In the phase of severe immunosuppression, viral reactivations or opportunistic infections occur frequently and account for the majority of all sepsis-related deaths, which can also occur years after hospitalization [2, 8]. Human leucocyte antigen-D related on monocytes (mHLA-DR) serves as a reliable biomarker for immune function and immunosuppression, since it correlates with immune competence, occurrence of nosocomial infections and mortality [2, 5, 8, 9].

Immune cell function is dependent on a 24-h circadian rhythm that is regulated by the expression of clock genes [10, 11]. Key immune cell types, macrophages and monocytes, have a robust intrinsic circadian clock and a high expression of clock genes [12]. Molecular circadian rhythms are generated by autoregulatory transcription–translation feedback loops [13]. The transcription factors *CLOCK* and *BMAL* induce the expression of their negative regulators *PER1-3* and *CRY1-2* [13]. The *PER–CRY* complex triggers its nuclear translocation and interacts with the *BMAL1–CLOCK* complex, which inhibits *PER* and *CRY* transcription [13]. When the expression of the PER–CRY complex decreases due to its proteasomal degradation, the cycle restarts [13].

Aberrant circadian rhythms have been associated with various pathological conditions resulting in lethal inflammation and immune dysfunction [14]. In addition, myocardial infarction, asthma and particularly inflammatory diseases show a day–night variation in appearance of symptoms and patients’ outcomes [15, 16]. In murine models, genetic ablation of the myeloid *Bmal1* gene results in an increased inflammatory response in experimental sepsis [14]. In sepsis patients, the severity of the inflammatory process is directly related to the degree of change in the clock genes *BMAL1* and *PER2* [17]. Coiffard et al. showed impaired circadian rhythms of cytokines, leukocytes and three clock genes (*BMAL1*, *PER2* and *PER3*) in severe trauma patients [18]. However, so far, no study has investigated the circadian system in critically ill patients with septic shock. Therefore, we tested whether expression of clock genes are disrupted in critically ill patients with septic shock.

## Patients and methods

### Study participants and data acquisition

This prospective observational study was performed at the university hospital Charité–Universitätsmedizin Berlin, Department of Anesthesiology and Operative Intensive Care Medicine (CCM, CVK). Between May 2014 and January 2018, we screened five anesthesiological or surgical ICUs, respectively, for patients with septic shock. All patients received guideline-based intensive care treatment in accordance to our standard operating procedures [19]. Our center’s policy requires nurses to turn off the light after dark, except when emergency procedures are required. Patients with septic shock and the need for norepinephrine dosages > 0.3 µg/kg/min for at least 2 h were included according to the American College of Chest Physicians (ACCP) consensus definition [20].

Age below 18 years, pregnant or lactating female patients, acute leukemia, severe leukocytosis (> 50.000/nL) or thrombocytopenia (< 5.000/nL), other immunosuppressing conditions (acute leukemia, organ transplantation, systemic prednisolone treatment, interferon therapy, chemotherapy or radiotherapy) or participation in an interventional study, acute pulmonary embolism or acute myocardial infarction within the last 72 h and cardiopulmonary resuscitation within the last 7 days were defined as exclusion criteria. The patient or legal guardian had to consent in the study participation. Primary outcome measure was the assessment of circadian expression of clock genes over 24 h. Secondary outcome measures were clinical scoring systems as Charlson Comorbidity Index (CCI), Sequential Organ Failure Assessment Score (SOFA), Simplified Acute Physiology Score II (SAPS II) and Therapeutic Intervention Scoring System (TISS-28), lactate, body temperature, time of mechanical ventilation, ICU and hospital length of stay and mortality.

**Fig. 1:**
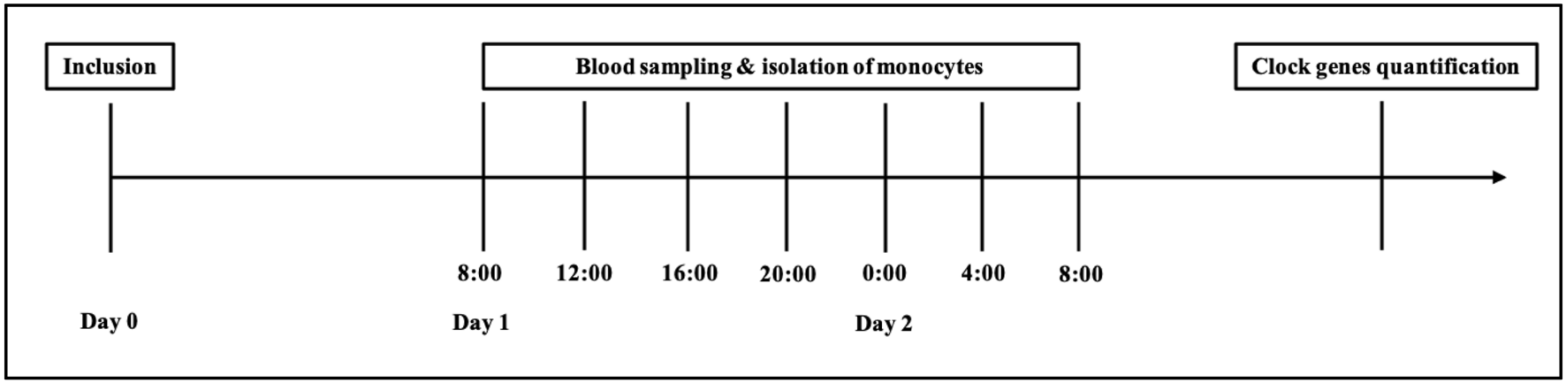
Study schedule from inclusion, over blood sampling and isolation of monocytes to gene quantification

For clock gene measurement, 5 mL blood in an EDTA vial and 5 mL blood in a serum vial were drawn each 4 h in 24 h starting the day after inclusion at 8:00 a.m. (Fig. 1). The core body temperature (CBT) was measured hourly using the indwelling urinary catheter. Clinical data and further laboratory values were taken from the charts for the 24-h period of clock gene measurement.

### Measurement of clock and clock-associated genes

The serum samples were centrifuged and the serum was conserved at − 80 °C. CD14-positive monocytes were isolated from the EDTA samples using an Auto-MACS Pro Separator (Miltenyi Biotec GmbH, Bergisch Gladbach). The isolated monocytes were stored at − 80 °C until transport.

A panel of 20 clock and clock-associated genes—*ARNTL*, *ARNTL2*, *BHLHE40*, *BHLHE41*, *CIART*, *CIPC*, *CLOCK*, *CRY1*, *CRY2*, *CSNK1D*, *CSNK1E*, *DBP*, *NFIL3*, *NPAS2*, *NR1D1*, *NR1D2*, *PER1*, *PER2*, *PER3*, *RORA—*were quantified using Nanostring™ PlexSet at each time point for each patient. Raw expression data were obtained using a NanoString nSolver Software (NanoString Technologies). A gene was considered expressed in a sample if its raw expression was greater than 3 times the background level (the geometric mean of the negative controls) for that sample. Then, technical normalization of the raw expression per sample was performed using the positive spike-in controls. Finally, the geometric mean of 4 housekeeping genes was used to normalize expression across samples followed by calibration of Nanostring probes across multiplexed samples (as per “Gene Expression Data Analysis Guidelines” from Nanostring). For a given patient, a clock gene time series was included if the gene was detected in at least 6 of the 7 time points. Only patients with at least 8 expressed clock genes were used for further study. Finally, a gene was retained in the final dataset only if it was expressed in at least 6 patients. The final dataset contained time series data from 17 patients on up to 15 genes. All expression data are *log*_*2*_ transformed in accordance with Nanostring™ analysis guidelines. The septic shock patients are labeled “SCXX”.

### Determination of circadian rhythms

The gene expression patterns of clock genes across all patients were subject to harmonic regression analysis with a fixed oscillation period of 24 h; very short time series (consisting of only 7 points herein) do not permit accurate estimation of periods. We characterized the amplitude of circadian rhythms in gene expression using confidence intervals (CI) (instead of a single estimate of amplitude). The CI represents the range of circadian amplitudes of gene expression that are consistent with the measurements at the 95% level (i.e., *p* < 0.05). The amplitude was defined as the fold-change in expression between the peak and trough of the rhythm.

It is generally understood that only transcript rhythms with a sufficiently large amplitude, defined as a greater than 1.4-fold (equal to 0.5 change in log_2_ scale) increase, are biologically relevant [21]. Using this threshold and the amplitude CIs, three classes of clock genes in the dataset could be defined. Clock genes with an amplitude CI falling completely above the threshold have biologically meaningful gene expression rhythms with adjusted-*p* < 0.05. Similarly, clock genes with amplitude CIs that fall completely within the threshold and zero have no rhythms with adjusted-*p* < 0.05 [22]. Finally, clock genes with amplitude CIs that include the threshold are inconclusive, i.e., the data are insufficient to determine rhythmicity or a lack thereof.

In order to better characterize the rhythmicity of the entire panel of clock genes in each patient, we defined a rhythmicity score ${S}_{P}$ which was defined as ${S}_{P}=\frac{{N}_{\mathrm{rhy}}-{N}_{\mathrm{arrhy}}}{{N}_{\mathrm{genes}}}$. ${S}_{P}$ is the difference between the fraction of genes that are rhythmic and the fraction of genes that are arrhythmic in that patient. The score ${S}_{P}$ takes a value 1, when all genes are rhythmic and a value -1 if all the genes are arrhythmic. To characterize the rhythmicity of a clock gene across patients, we defined a rhythmicity score for a clock gene ${S}_{G}$ similar to the one above, but the averaged value across the number of patients it is observed in, i.e., ${S}_{G}=\frac{{N}_{\mathrm{rhy}}-{N}_{\mathrm{arrhy}}}{{N}_{\mathrm{patients}}}$.

### Data from body time study (BOTI)

To determine whether the circadian clocks in septic shock patients are different from healthy young adults, we compared clock genes from our study against clock genes from the BOTI study [23]. We used gene expression time series measured from CD14 + monocytes of 12 healthy young male subjects kept under constant routine using Nanostring™ between May 2015 and Jan 2016; details on the study participants and design can be found in Wittenbrink et al. [23]. Subjects in this study were sampled every 3 h resulting in 9 samples over 24 h. Therefore, we dropped two time points 12 h apart in order to have the same 7 samples over 24 h as in septic shock patients. This was important, since the computed amplitude confidence intervals (CI) also incorporate information on the number of time points available to estimate amplitude. Furthermore, only 7 clock genes—*PER1*, *PER2*, *NR1D1*, *NR1D2*, *DBP*, *CRY1*, *CRY2*—were measured in common between the two studies. Therefore, we restricted our comparisons of the two studies to these 7 clock genes. The subjects from the BOTI study are labeled “BXX”.

We first calculated CI for the clock genes in BOTI subjects, which were then compared to septic shock patients. We next computed rhythmicity scores for each septic shock patient and for each BOTI subject based on only the 7 common clock genes. We compared the rhythmicity of the common clock genes in septic shock patients and BOTI subjects using rhythmicity scores.

### Measurement of HLA-DR

The expression of mHLA-DR on monocytes was measured using flow cytometric measurement and was taken from routine laboratory results [24]. The time point of mHLA-DR measurement was the day of inclusion; if not available, we took the closest measurement from the charts.

### Statistical analysis

The data were analyzed using R statistical software (ver. 3.6.2) and SPSS 26.0 (IBM Corporation, Armonk, NY). The amplitude CI for each gene and subject was computed as follows: using the *lm* function, we regressed the data with the function $m+{b}_{1}\mathrm{cos}\left(2\pi \frac{t}{24}\right)+{b}_{2}\mathrm{sin}(2\pi \frac{t}{24})$ to obtain the best fit and the residuals. Bootstrap data were generated by adding the best fit to sampled residuals (with replacement) [25]. Regressing our harmonic function to the bootstrap data yielded bootstrap estimate of amplitude ($A=\sqrt{{b}_{1}^{2}+{b}_{2}^{2}}$), we performed 3999 bootstrap repetitions (using R-package ‘boot’ (v.1.3–24)). The 95% CI for amplitude was computed from the distribution of bootstrap estimates after Bonferroni correction to account for multiple gene testing within each patient; multiple testing correction widened the CIs (Additional file 1: Table S1). Results are expressed in medians ± quartiles or percentage, respectively. The level of statistical significance for all analyses was 0.05.

## Results

### Study population

**Fig. 2:**
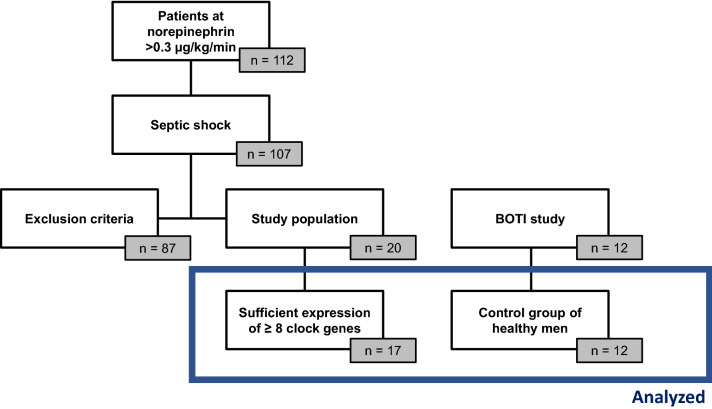
CONSORT diagram, 17 patients were included to the final analyses

During the study period, 20 of the 112 patients screened were included (Fig. 2). Of these, 3 patients were excluded from further analysis, since their samples expressed fewer than 8 clock genes. Our final analysis cohort thus contained 17 patients. The basic patient characteristics and outcome parameters are shown in Table 1.

**Table 1:** Patient characteristics and secondary outcome parameters

Parameter	Value (*N* = 17)
Age (years)	66 (51.5–74)
Male sex, *n* (%)	7 (41.2)
BMI (kg/m^2^)	27.7 (23.6–33.4)
TISS-28	42 (37–49.5)
CCI	6 (5–8)
SOFA	17 (11.5–17.5)
SAPS II	69 (57–79.5)
Hemoglobin (g/dL)	8.6 (7.9–10)
Leukocytes (/nL)	14.8 (8.7–19.5)
INR	1.31 (1,27–1.53)
Thrombocytes (/nL)	104 (65–218)
mHLA-DR (antigens/cell)^a^	5632 (3322–10,192)
Lactate (mg/dL)	15 (12–42.5)
PTT (sec)	40.6 (37.5–57.9)
Hemodialysis, *n* (%)	14 (82.4)
ECMO, *n* (%)	3 (17.6)
Ventilation *n* (%)	17 (100)
Time of mechanical ventilation (hours)	471 (331–910)
ICU length of stay (days)	25 (15.5–40)
Hospital length of stay (days)	29 (15.5–42.5)
In-hospital mortality, *n* (%)	7 (41.2)

Values at the first point of clock genes measurement, continuous quantities in median with quartiles

*BMI* body mass index, *CCI* Charlson Comorbidity Index, *ECMO* extracorporeal membrane oxygenation, *ICU* intensive care unit, *INR* International Normalized Ratio, *mHLA-DR* human leucocyte antigen-D related on monocytes, *PTT* partial thromboplastin time, *SAPS II* Simplified Acute Physiology Score II, *SOFA* Sequential Organ Failure Assessment Score, *TISS* Therapeutic Intervention Scoring System

^a^mHLA-DR was measured in 14 patients and median 0.5 (− 3.25–0.25) days before inclusion

### Mixture of rhythmic and arrhythmic genes in septic shock patients

**Fig. 3:**
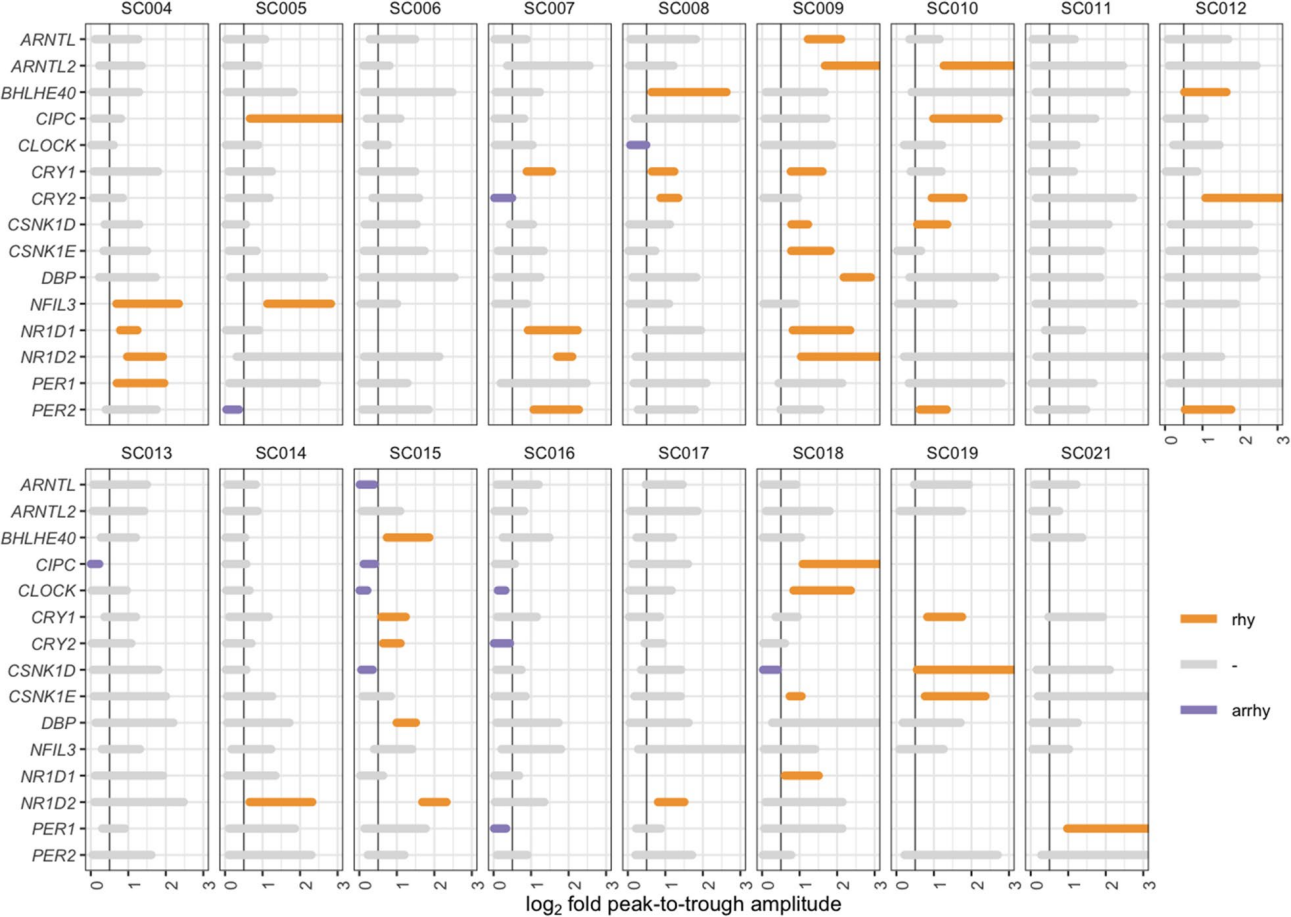
Quantification of rhythmicity of clock genes in septic shock patients. Confidence intervals (CI) on the rhythm amplitudes (peak to trough on the log_2_ scale) of gene expression based on harmonic regression and bootstrapping are visualized as horizontal bars. The CIs include the true amplitude of the clock gene with a 95% probability. The color of the bars indicates whether the rhythms are present (orange), absent (violet) or data are inconclusive (grey). The vertical line shows the minimum amplitude that is deemed biologically relevant. Bars are missing for certain clock genes that did not satisfy the inclusion criteria

The gene expression patterns of clock genes across all patients are shown in Additional file 2: Figure S1. Classification of clock genes in the dataset revealed a complex picture (Fig. 3). Some patients (e.g., SC009 and SC011) had robust rhythms in several clock genes, while others (e.g., SC013 and SC016) showed a few arrhythmic clock genes and no conclusively rhythmic genes. The gene expression profiles (Additional file 2: Figure S1) are color-coded to reflect this classification of the clock genes. About 75% of the measured clock genes were classified as having inconclusive rhythms. This suggests that either many clock genes indeed have dampened rhythms or that the number of measurements is insufficient to obtain enough statistical power.

The rhythmicity scores for the septic shock patients were ${S}_{P}=0.15\pm 0.18$ (Fig. 2). The clustering of the rhythmicity score close to zero reflects the large fraction of clock genes classified as inconclusive. Moreover, the few patients with negative scores have more arrhythmic clock genes than rhythmic ones.

We also tested whether the rhythmicity scores ${S}_{P}$ were associated with demographic or secondary clinical outcome parameters of the septic shock patients. However, we could not identify any statistically significant linear association or correlation between the demographic (age, gender, BMI) and secondary outcome parameters (SAPS II, SOFA, TISS-28, lactate, body temperature, time of mechanical ventilation, ICU length of stay, hospital length of stay, mortality) and the rhythmicity score.

Core body temperature (a secondary outcome parameter) is regulated by the central circadian clock and shows circadian rhythms with an amplitude of about 0.5 °C in healthy humans. The core body temperature of septic shock patients showed strong trends with 7 patients each having increasing and decreasing trends (Additional file 3: Figure S2A). On detrending, core body temperature did show variation of the order of 0.5° C or more, but with no stable 24-h rhythmicity and minima at different times of the day (Additional file 3: Figure S2B).

### Specific clock genes are rhythmic (or arrhythmic) across septic shock patients

**Fig. 4:**
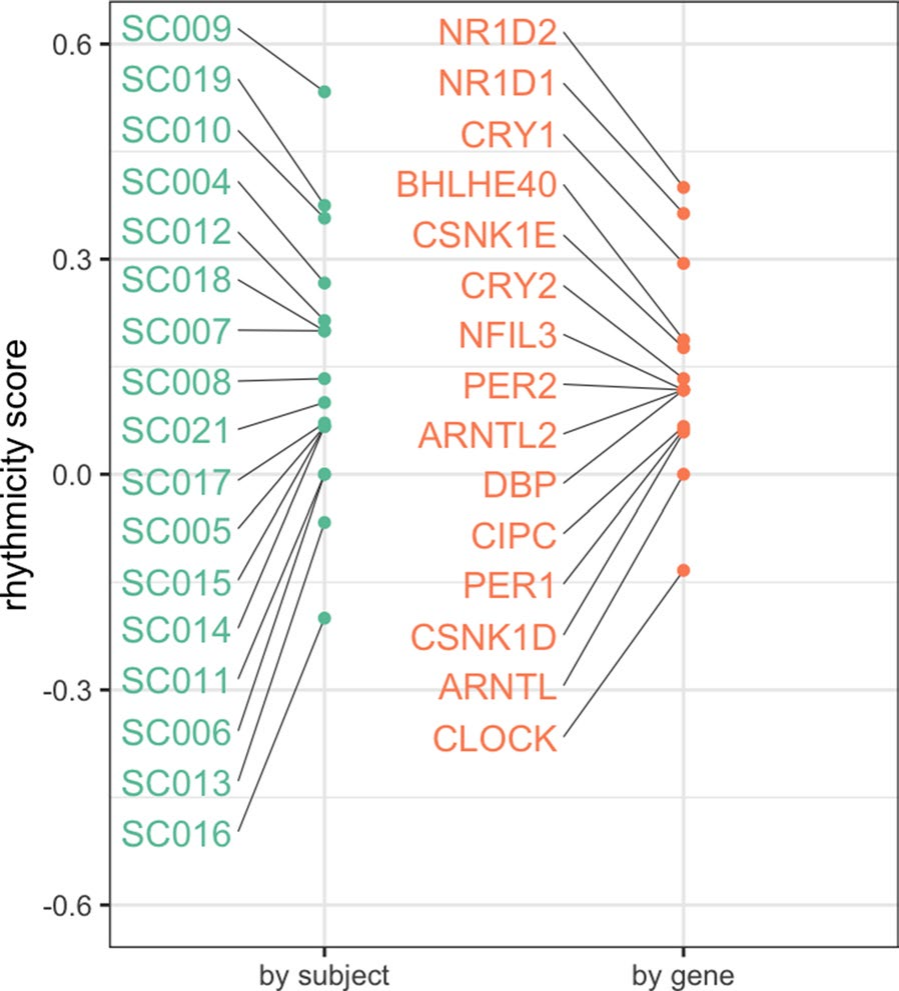
Rhythmicity of clock genes in septic shock patients grouped by patient and clock gene. The rhythmicity score takes a value between − 1 and 1, with a value of 1 and − 1 representing rhythmicity and arrhythmicity, respectively. The scores shown are aggregates of all clock genes in a subject (‘by subject’), or of all subjects for a particular clock gene (‘by gene’)

We next enquired whether specific clock genes were more or less rhythmic across the set of septic shock patients. The rhythmicity scores for the clock genes were clustered similarly to the rhythmicity score of the patients, ${S}_{G}=0.15\pm 0.1$4 (Fig. 4). The clock genes *NR1D1, NR1D2* and *CRY2* were the most rhythmic across patients, while *CLOCK* and *ARNTL* were the least rhythmic (with non-positive scores). The high score of *NR1D1* must be regarded here with caution as reliable expression was detected in only 11 out of the 17 patients. In summary, the positive regulators of the transcriptional–translation feedback loop [26] (*CLOCK* and *ARNTL*) were the least rhythmic, while negative regulators (*NR1D1*, *NR1D2*, *CRY2*) were the most rhythmic in septic shock patients.

### Clock gene expression levels are altered in septic shock patients

**Fig. 5:**
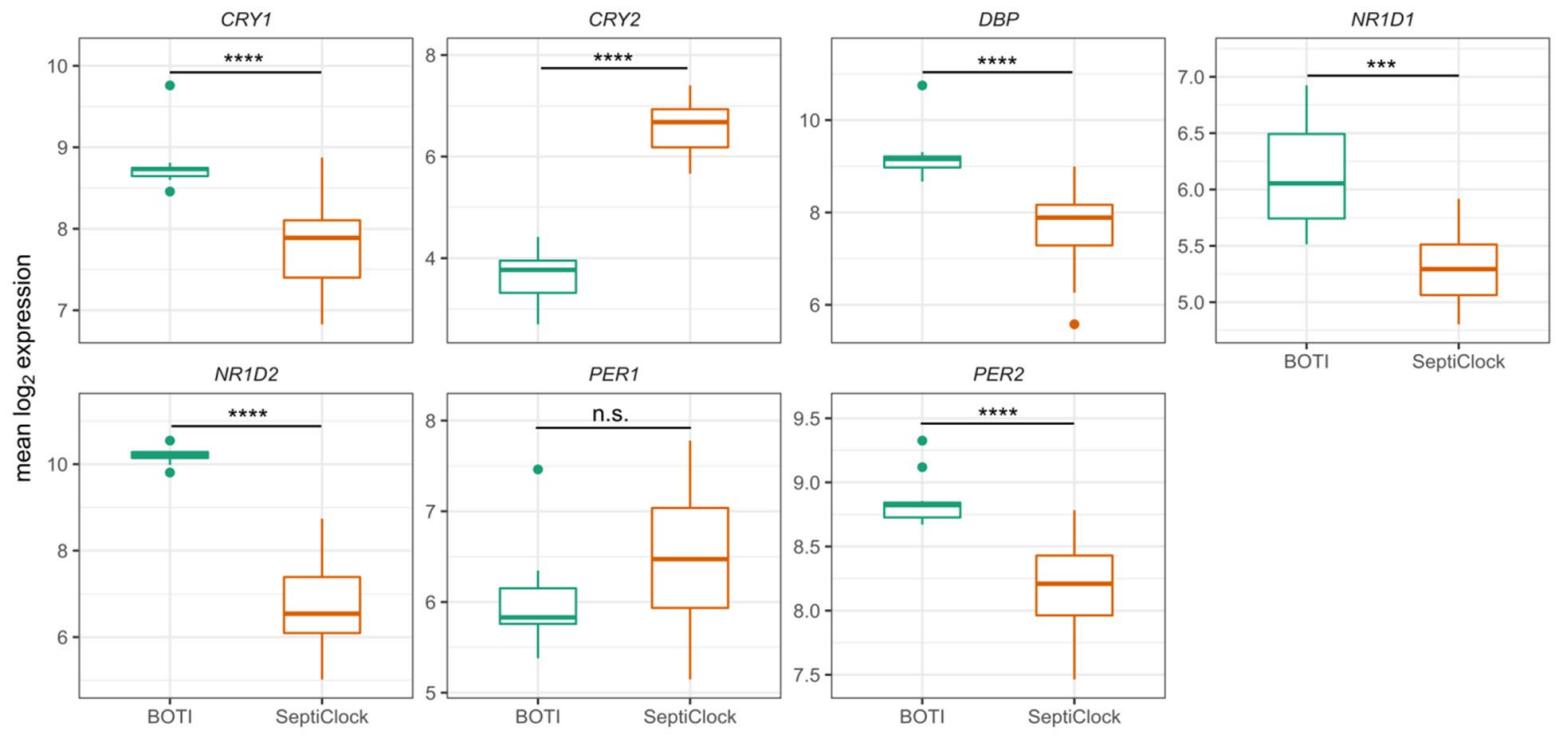
Comparison of average clock gene expression of the 7 common clock genes in septic shock patients and BOTI subjects. The log_2_ gene expression average across time points for each septic shock patient (*N* = 17) in the septic shock patients and healthy young men (*N* = 12) from the BOTI study. P-values for the significance of the difference are based on the non-parametric Wilcoxon rank-sum test. ****p < 0.0001, ***p < 0.001, *n.s.* *p* > 0.05

We first compared mean expression levels of the clock genes to determine if clock gene expression was altered in septic shock patients. Six of the 7 clock genes showed significantly altered average expression in septic shock patients compared to healthy young men from the BOTI study (Fig. 5). While *CRY1*, *NR1D1*, *NR1D2*, *DBP*, *PER2* were suppressed in septic shock patients, *CRY2,* surprisingly was significantly upregulated compared to healthy young men. Moreover, the most differentially expressed clock genes were *NR1D2* and *CRY2*, which were sevenfold down-regulated and 12-fold upregulated, respectively.

### Clocks of septic shock patients are less rhythmic than of healthy young adults

**Fig. 6:**
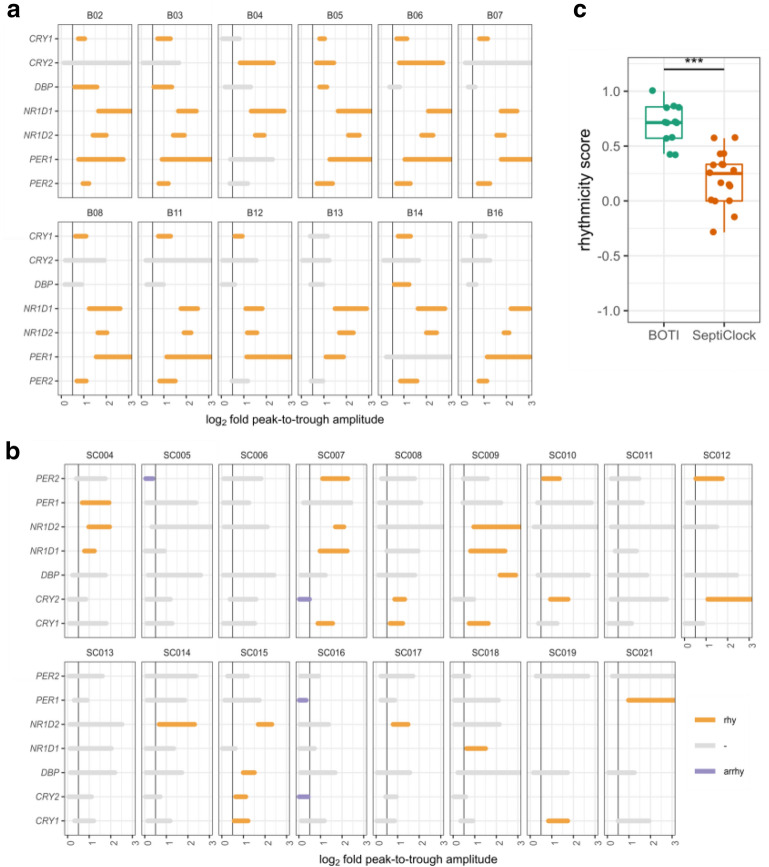
Comparison of rhythmicity of common clock genes between septic shock patients and healthy young men. **a** The confidence intervals (CI) for the clock genes from BOTI subjects used to classify clock genes as rhythmic, arrhythmic or inconclusive (similar to Fig. 2). **b** The confidence intervals for the common clock genes in septic shock patients extracted from Fig. 2 for comparison with (**a**). **c** The comparison of the rhythmicity scores between septic shock patients and healthy young adults based on the classification in (**a**, **b**). The scores were compared using a non-parametric Wilcoxon rank-sum test

CI for the clock genes in BOTI subjects are shown in Fig. 6a. In comparison to septic shock patients, most of the 7 clock genes were classified as robustly rhythmic in all BOTI subjects (Fig. 6b, Additional file 4: Figure S3). We found the rhythmicity scores for septic shock patients (0.23 ± 0.26) were lower (one-sided Wilcoxon rank-sum test, *p* < 10^–4^) compared to healthy young men (0.70 ± 0.18) (Fig. 6c). Nevertheless, some caution is warranted as the two groups were not age-matched and the scores were calculated using a small set of clock genes.

## Discussion

In this prospective observational pilot study, we examined the rhythmicity of the 15 most common clock and clock-associated genes in 17 patients with septic shock. The positive regulators *CLOCK* and *ARNTL (BMAL1)* had the lowest rhythmicity score and were most arrhythmic, while the negative regulators *NR1D1*, *NR1D2* and *CRY2* were the most rhythmic. In comparison to healthy young men, a decreased mean expression of almost every clock gene in patients with septic shock was shown, except for *CRY2*, which was upregulated. No association with secondary clinical parameters was found.

Other studies also investigated circadian rhythms in critically ill patients. Coiffard et al. included 38 severe trauma patients, of whom at least 13 patients developed a septic episode [18]. All trauma patients had disrupted rhythms of cortisol, leukocytes and cytokines. Among the three measured clock genes *BMAL1*, *PER2* and *PER3* only the last two were down-regulated. Diaz et al. investigated circadian rhythms in 11 patients in neurological intensive care units [27]. On the day of admission, all patients had a rhythmic expression of *CLOCK*, *BMAL1*, *CRY1* and *PER2*, whereas after one week the clock gene *CLOCK* showed a disrupted circadian rhythm [27]. A recent study of Acuna-Fernandez et al. compared *BMAL1*, *CLOCK*, *PER2* and *CRY1* expression in healthy volunteers, ICU and sepsis patients [17]. In line with our results, the authors found disrupted circadian rhythms in sepsis patients. In addition, they showed that conditions in ICUs, such as permanently high light levels, do not disrupt circadian rhythms in sepsis patients, because non-sepsis ICU-patients expressed clock genes rhythmically. In comparison to these studies, we investigated a larger panel of clock genes and were able to show a down-regulation of *BMAL1* and *PER2* as well. Additionally, we found that *NR1D1*, *NR1D2*, *CRY1*, *CRY2*, *PER1*, *PER2* and *DBP* had altered rhythms in at least some patients with septic shock.

Secondary outcome parameters including SOFA, SAPS II and TISS-28, lactate, time of mechanical ventilation, ICU and hospital length of stay and mortality did not show a linear correlation with rhythmicity scores. The core body temperature measurements coincident with clock gene measurements were highly variable with strong trends and therefore, could not be associated with clock gene expression amplitudes. Core body temperature varies rhythmically with its peak in the evening and trough in the morning hours [16]. However, the core body temperature troughed at different times during the day in different patients, which is in agreement with our finding of disrupted circadian rhythmicity. Generally, due to the small number of patients, associations of rhythmicity scores with outcome parameters might have gone undetected. Larger studies should examine whether disrupted rhythmicity is associated with mortality.

As another clinical secondary outcome parameter, we investigated the correlation between circadian rhythmicity and the expression of mHLA-DR in monocytes, which is one of the most studied markers of immunosuppression. A decreased mHLA-DR expression is associated with higher mortality in critically ill as well as sepsis patients [28]. Due to the robust intrinsic circadian rhythm of monocytes [11], we hypothesized that in patients with septic shock the grade of immunosuppression as measured by mHLA-DR expression is associated with a loss of circadian rhythm. In our study population, 11 out of 14 patients had levels of HLA-DR of 15.000 molecules per monocyte or less, which is associated with immunosuppression and immunoparalysis [24]. However, HLA-DR expression on monocytes did not show an association or significant correlation with the rhythmicity score. Nearly all patients showed immunosuppression and no rhythmicity in the expression of some clock genes. This could be attributed to the small study population not capturing the multitude of factors influencing clock gene expression.

It is recognized that melatonin helps restore and maintain circadian homeostasis [29]. Additionally, melatonin was reported to have immunomodulatory and antioxidative effects [30]. Lorente et al. found that melatonin levels in non-surviving sepsis patients were higher than in survivors. The authors suggest that the higher serum melatonin levels in patients who did not survive are due to an attempt to avoid a dangerous situation; however, these higher serum melatonin levels in non-survivors are not sufficient to definitively compensate for the situation [31]. Melatonin is used as a supporting drug in septic infants and could be a promising therapeutic option in sepsis patients to promote stable circadian rhythms, improve immunity and outcomes [32].

There is a lack of evidence concerning the impact of circadian rhythmicity on pharmacodynamics and medications. Few studies suspect correlations between altered circadian rhythms and catecholamines or sedation [33]. However, further research is needed to analyze the impact of the circadian rhythm on sepsis mortality and its translational potential to find promising treatment options for improving sepsis care [34]. Understanding circadian rhythmicity in sepsis and the implications from and on the immune response are fundamental to develop new diagnostic and therapeutic tools and to individualize sepsis treatment.

This study has several limitations. First, only septic shock patients were included in our study. Thus, no conclusion can be drawn as to whether the disruption of the circadian rhythm favors sepsis or whether sepsis is the cause of the arrhythmia. Second, the inability of the analysis to classify clock genes as rhythmic or arrhythmic was due to a limited number of samples in the time series, i.e., we measured only 7 time points for each subject. When the rhythms are partially disturbed as is the case here, more data points are needed to detect arrhythmicity than to show rhythmicity. Third, the differences in mean expression levels of clock genes can both cause the changes in rhythmicity observed in septic shock patients and affect our statistical ability to quantify rhythms. Fourth, the clock genes measurement took place at different time points after hospitalization, in different stages of sepsis and only at one time point in the septic shock episode. Fifth, cortisol levels were not measured, hence this parameter, which plays an important role in the maintenance of circadian rhythmicity, cannot be used for comparison with other studies. Finally, samples from septic shock patients and BOTI patients were measured using slightly different Nanostring™ technologies, which—although unlikely—may result in differences in quantified expression levels.

## Conclusion

In conclusion, our study showed impaired circadian rhythmicity in patients with septic shock. The positive regulators of the transcriptional–translation feedback loop (*CLOCK* and *ARNTL*) were the least rhythmic, while negative regulators (*NR1D1*, *NR1D2*, *CRY2*) were the most rhythmic. In addition, the expression of the clock genes *CRY1*, *NR1D1*, *NR1D2*, *DBP*, and *PER2* were suppressed in septic shock patients, whereas *CRY2* was significantly upregulated compared to healthy young men. These results strengthen the relationship between circadian disruption, a restricted immune response, and sepsis.

## Supplementary Information

**Additional file 1: Table S1**. Confidence intervals per clock gene and subject, representing the range of circadian amplitudes of gene expression that are consistent with the measurements at the 95% level (i.e., *p* < 0.05). The amplitude was defined as the fold-change in expression between the peak and trough of the rhythm. Rhythmic genes are marked as rhy, arrhythmic genes as arrhy, respectively.

**Additional file 2: Figure S1**. Gene expression patterns for all clock genes quantified in septic shock patients. The lines are colored according to the classification of rhythmicity in Fig. 2. The empty boxes represent clock genes that did not pass the inclusion criteria for the analysis.

**Additional file 3: Figure S2**. Core body temperature time series of the septic shock patients. Core body temperature was measured every hour during a 24-h period during the blood draw. The raw time series in (A) was linearly detrended (trend is shown in blue) to obtain (B).

**Additional file 4: Figure S3.** Gene expression patterns for the clock genes measured in the BOTI study that were also measured in septic shock patients (compare with Additional file 2: Figure S1). The lines are colored according to the classification of rhythmicity in Fig. 6a.

## Data Availability

Due to legal restrictions imposed by the Ethics Committee and the data protection commissioner of the Charité–Universitätsmedizin Berlin, public sharing of study data with other researchers or entities is not allowed. Requests may be sent to dai-researchdata@charite.de.

## Abbreviations

ACCP: American College of Chest Physicians
BMI: Body mass index
CARS: Compensatory anti-inflammatory response syndrome
CBT: Core body temperature
CCI: Charlson Comorbidity Index
CI: Confidence interval
ECMO: Extracorporeal membrane oxygenation
ICU: Intensive care unit
INR: International Normalized Ratio
mHLA-DR: Human leucocyte antigen-D related on monocytes
PTT: Partial thromboplastin time
SAPS II: Simplified Acute Physiology Score II
SIRS: Systemic inflammatory response syndrome
SOFA: Sequential Organ Failure Assessment Score
TISS-28: Therapeutic Intervention Scoring System
