# Supplementary figures and images for: Circadian rhythms in septic shock patients
Source: Ann Intensive Care. 2021 Apr 26;11:64. doi: 10.1186/s13613-021-00833-5 (PMC8076360; doi:10.1186/s13613-021-00833-5)

## Slide 1
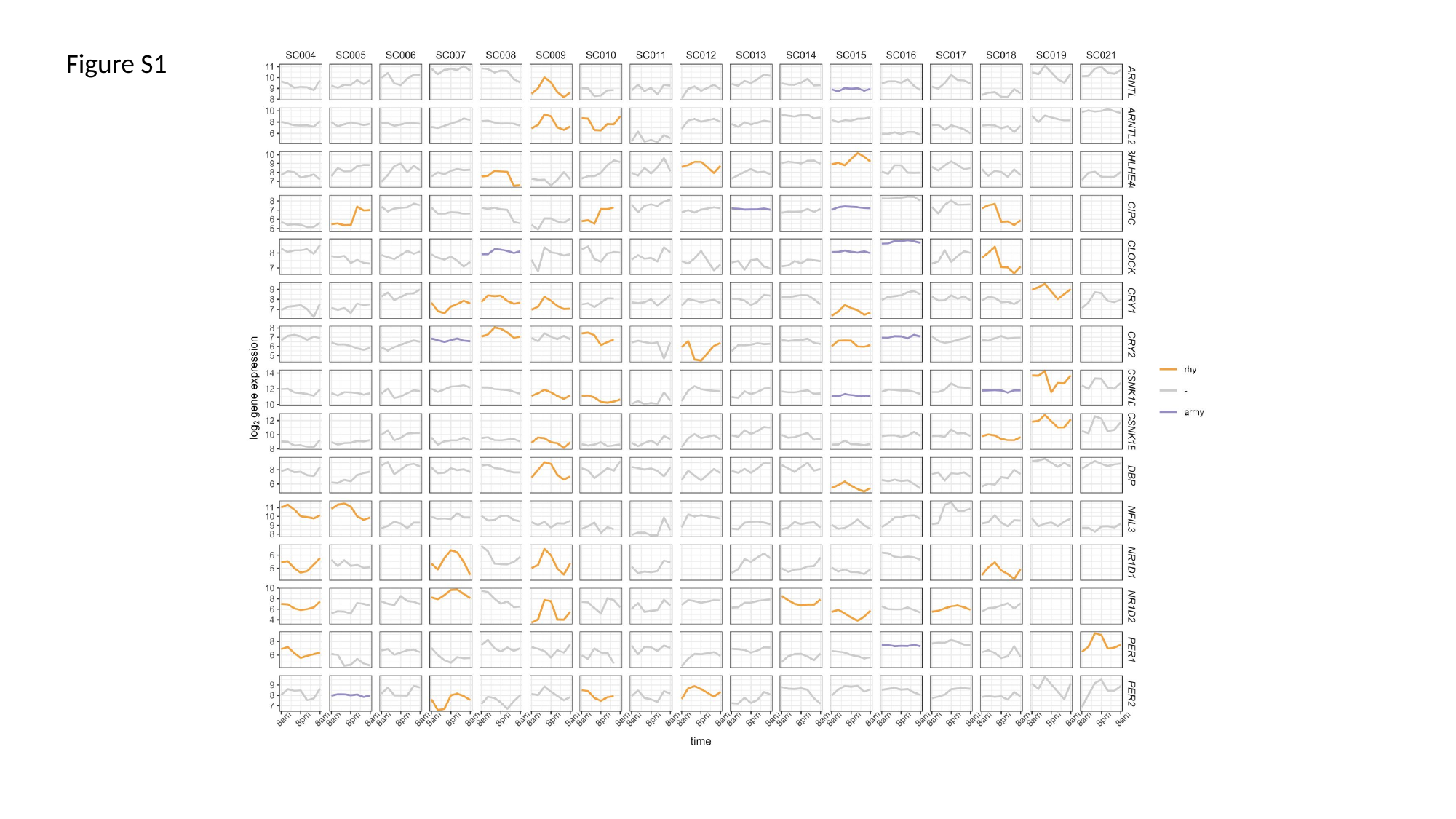

Figure S1

Supplement: Supplementary file 2 — Additional file 2: Figure S1. Gene expression patterns for all clock genes quantified in septic shock patients. The lines are colored according to the classification of rhythmicity in Fig. 2. The empty boxes represent clock genes that did not pass the inclusion criteria for the analysis. [file 13613_2021_833_MOESM2_ESM.pptx]

## Slide 1
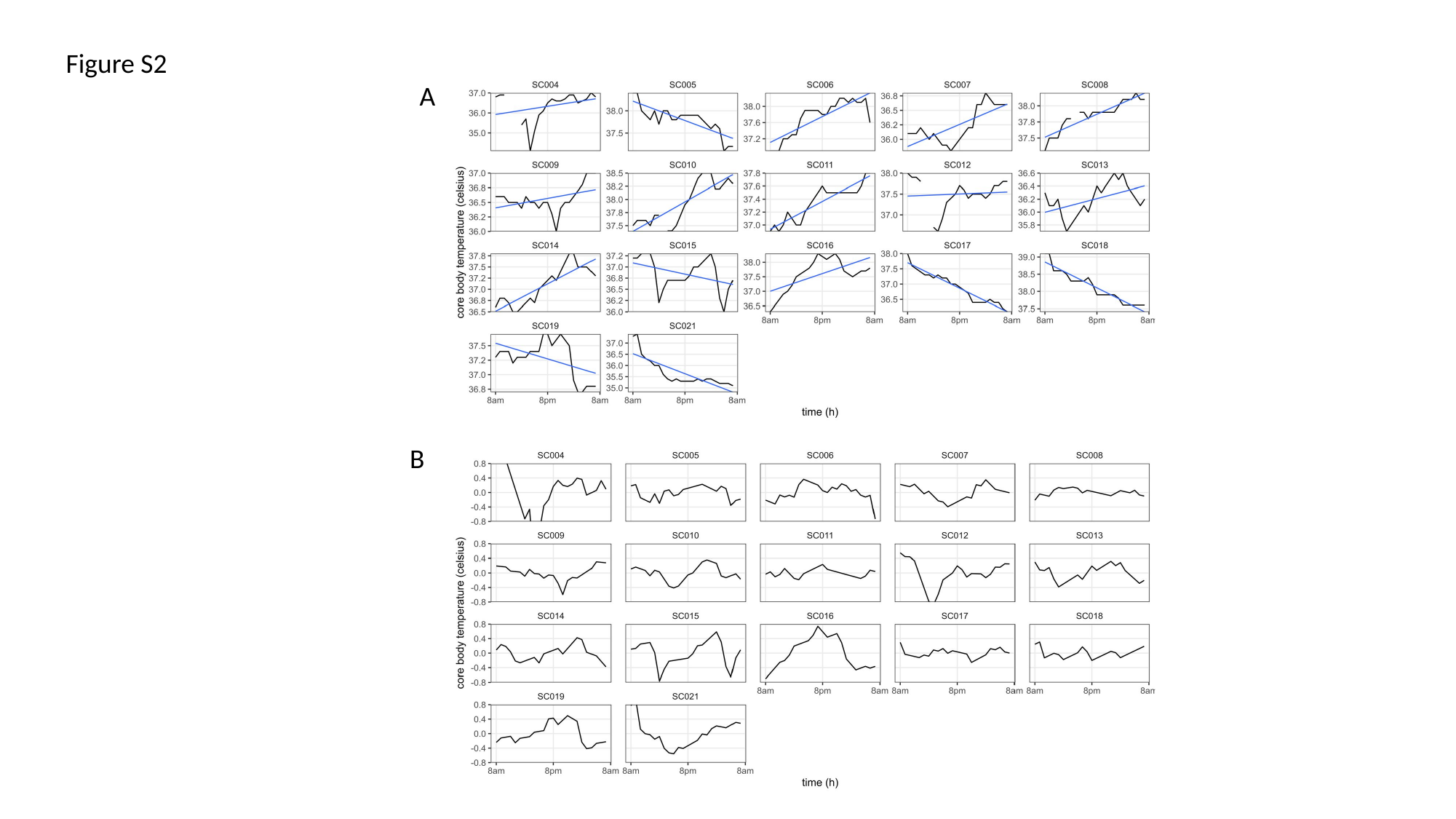

Figure S2
A
B

Supplement: Supplementary file 3 — Additional file 3: Figure S2. Core body temperature time series of the septic shock patients. Core body temperature was measured every hour during a 24-h period during the blood draw. The raw time series in (A) was linearly detrended (trend is shown in blue) to obtain (B). [file 13613_2021_833_MOESM3_ESM.pptx]

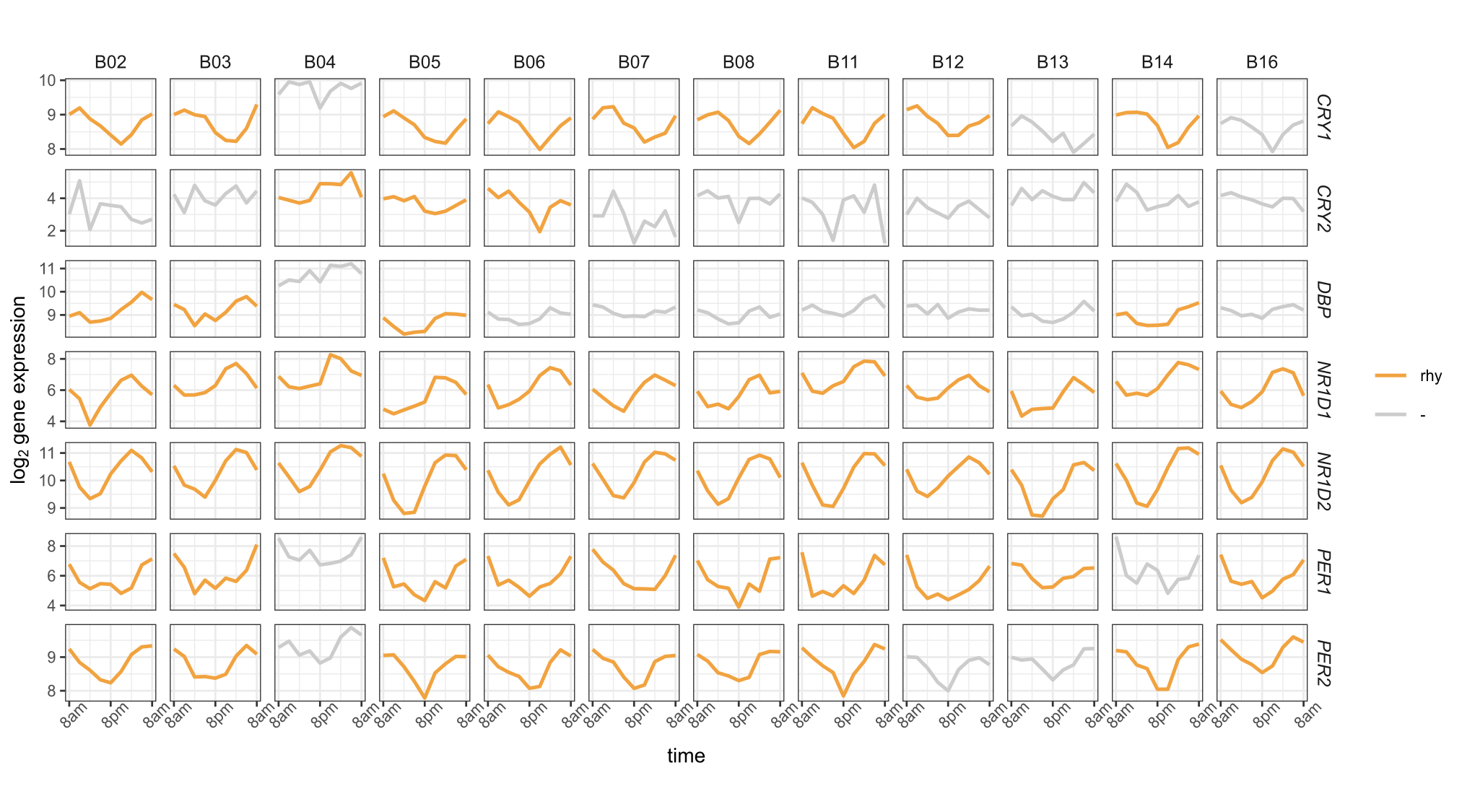

Supplement: Supplementary file 4 — Additional file 4: Figure S3. Gene expression patterns for the clock genes measured in the BOTI study that were also measured in septic shock patients (compare with Additional file 2: Figure S1). The lines are colored according to the classification of rhythmicity in Fig. 6a. [file 13613_2021_833_MOESM4_ESM.png]
